# Warfarin use and vestibular dysfunction insights from NHANES data, network pharmacology, Mendelian randomization, and molecular docking

**DOI:** 10.1038/s41598-025-96681-5

**Published:** 2025-04-06

**Authors:** Shihan Liu, Lingli Zhang, Wenlong Luo

**Affiliations:** 1 Department of Otorhinolaryngology, The Second Affiliated Hospital of Chongqing Medical University, Chongqing, China; 2 Department of Otorhinolaryngology, Central Hospital Affiliated to Chongqing University of Technology, Chongqing, China

**Keywords:** Warfarin, Vestibular dysfunction, Network Pharmacology, Mendelian randomization, MAPK8, Neurology, Pharmacology

## Abstract

Despite numerous anticoagulants available, warfarin is widely used due to its efficacy and cost-effectiveness in treating thromboembolic diseases. However, its potential impact on vestibular function remains unexplored. This study investigates the association between warfarin use and vestibular dysfunction using data from the NHANES database and examines underlying mechanisms through network pharmacology, Mendelian randomization (MR), and molecular docking. We conducted a cross-sectional analysis of NHANES data (1999–2004) to evaluate the prevalence of vestibular dysfunction among warfarin users. Network pharmacology identified overlapping genes between warfarin targets and vestibular dysfunction-related genes. MR analysis assessed the causal relationship, and molecular docking examined interactions between warfarin and significant genes. The study included 1681 participants, revealing a higher prevalence of vestibular dysfunction in warfarin users. Multiple regression analysis confirmed a significant association between warfarin use and vestibular dysfunction. Network pharmacology identified 31 overlapping genes, with MAPK8 emerging as a key gene through MR analysis. Molecular docking showed a strong binding affinity between warfarin and MAPK8. Findings suggest that warfarin use is significantly associated with vestibular dysfunction, potentially through interactions with MAPK8. This highlights the importance of monitoring vestibular function in patients on warfarin therapy and considering genetic factors to personalize treatment. Future research should explore these mechanisms further and validate findings in broader populations.

**Supplementary Information:**

The online version contains supplementary material available at 10.1038/s41598-025-96681-5.

## Introduction

Although there are currently many anticoagulants available, warfarin remains widely used as an oral anticoagulant for the prevention and treatment of thromboembolic diseases due to its significant efficacy and cost-effectiveness^1^. These diseases include atrial fibrillation, deep vein thrombosis, and pulmonary embolism^2,3^. However, like any medication, warfarin is associated with potential risks and adverse effects, some of which can affect patients’ quality of life and adherence to treatment, such as an increased risk of bleeding^4,5^.

Current research on warfarin-related complications primarily focuses on hemorrhagic complications, but there is growing evidence suggesting that warfarin use may also affect cognitive function^6,7^ and increase the risk of Alzheimer’s disease^8,9^. Vestibular function, to some extent, predicts changes in cognitive function^10–12^. Dobbels et al. found that patients with acquired bilateral vestibulopathy exhibited cognitive impairments, suggesting a link between vestibular dysfunction and cognitive decline^10^. Jacob et al., in their study of an aging population, discovered that vestibular function is associated with alterations in cortical and sub-cortical structures, which may impact cognitive function^11^. Additionally, Smith noted that the vestibular system is closely connected to cognitive function, with damage to the vestibular system potentially leading to cognitive impairments^12^. The possible mechanism between vestibular function and cognitive function is that the vestibular system has extensive neural connections with various brain regions involved in regulating cognitive function. Vestibular inputs may affect cognitive processes such as attention, spatial perception, and memory^13^. Given the relationship between vestibular and cognitive functions, as well as the potential impact of warfarin on the nervous system, we hypothesize that the use of warfarin may affect cognitive function by influencing vestibular function, or changes in vestibular function may be a potential biomarker for warfarin - induced cognitive - related complications. Therefore, studying the impact of warfarin on vestibular function will not only help to understand the potential side effects of this drug, but may also provide a new perspective for early detection and prevention of cognitive disorders.

Given the potential impact of warfarin on cognitive function and the known relationship between vestibular and cognitive function, we hypothesize that the use of warfarin is associated with vestibular dysfunction. Specifically, we investigate the association between warfarin and vestibular dysfunction through the National Health and Nutrition Examination Survey(NHANES) database, and explore the causal relationship between the two using Mendelian randomization(MR). Subsequently, we propose and investigate that warfarin may affect vestibular function through interaction with key genes (such as MAPK8), which are involved in the drug’s mechanism of action and the regulation of vestibular function.

This study intends to conduct a cross-sectional analysis of the potential correlation between warfarin use and the prevalence of vestibular dysfunction in adults using the NHANES database. Additionally, we will use network pharmacology, MR, and molecular docking methods to deeply investigate the mechanisms by which warfarin may affect vestibular function. Our aim is to provide clinicians with deeper insights into the relationship between warfarin use and vestibular health. NHANES is an ongoing, nationally representative research program designed to monitor the health and nutritional status of the US population. Network pharmacology is used to identify key genes involved in drug-disease interactions^14^. MR is a statistical method used to explore causal relationships between diseases or between targets and diseases^15^. Molecular docking studies the interactions between molecules and predicts their binding modes and affinities^16^.

## Methods

**Fig. 1:**
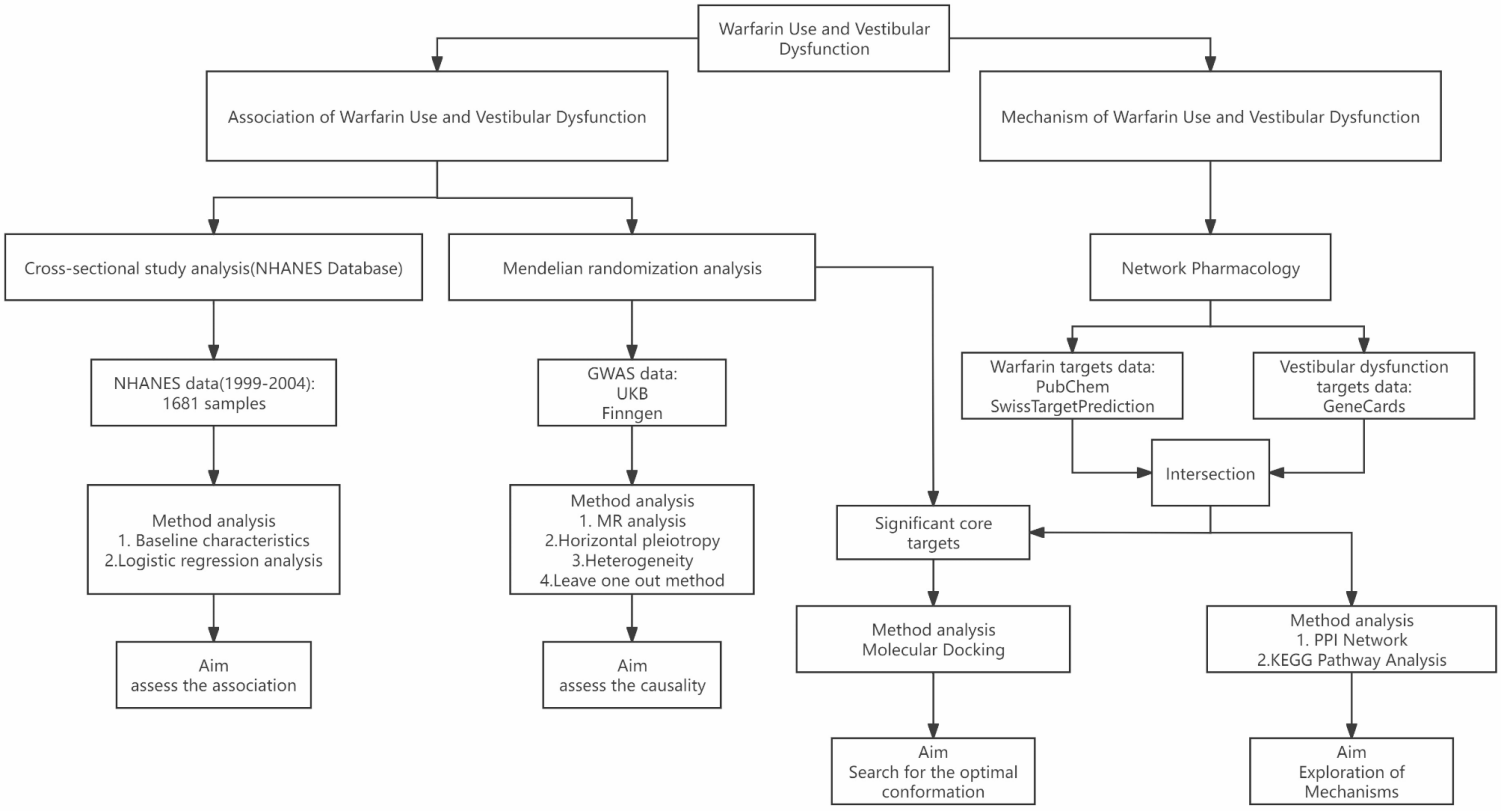
Flow chart of this study.

This study explored the relationship and potential mechanisms between warfarin ues and vestibular dysfunction through a multi-stage approach. First, we investigated the association between warfarin use and vestibular dysfunction using the NHANES database. Subsequently, we screened for genes potentially involved in warfarin-induced vestibular dysfunction through network pharmacology. We further validated the possible causal relationship using MR and identified significant genes potentially affected by warfarin. Finally, we performed molecular docking to validate the interaction between warfarin and the identified significant genes.(Fig. 1).

### NHANES database analysis

#### Data source and study population

NHANES is a national research program that provides representative data on the health and nutritional status of the US population. The National Center for Health Statistics’ Ethics Review Board approved all study protocols, and informed consent was obtained from all participants for the public data. Our study used NHANES data from 1999 to 2004 because only this period included the Romberg test. All data were obtained from the official website. The inclusion criteria were participants who completed the tests and questionnaires, excluding those who did not use warfarin or lacked Romberg test and covariate data.

#### Prescription drug use

Participants’ exposure to warfarin was determined based on their responses to the questions in the prescription drug use section: “Have you taken or used any prescription drugs in the past month?” .The answer “no” was defined as the control group, and those who had used warfarin were defined as the exposed group.

#### Vestibular dysfunction

Vestibular dysfunction diagnosis relied on participants completing the Romberg test, available in the NHANES database during 1999–2004. The Romberg test assessed the subject’s ability to maintain balance across four progressively challenging conditions. Failure in any test condition denoted vestibular dysfunction, while successful completion of all conditions indicated normal vestibular function^17,18^.

The Romberg test was used to examine the ability of the subject to stand independently in four test conditions with progressively increasing difficulty. Condition 1: Subjects stood using all sensory inputs that contribute to balance -central vestibular system, vision, and proprioception (sense of leg muscle position). Condition 2: Subjects were tested for balance in a condition in which only vestibular and proprioceptive information was provided (subjects closed their eyes to eliminate visual input). Condition 3: Subjects were balanced on a foam-filled surface for evaluation of visual and vestibular function. Condition 4: Subjects closed their eyes (visual input was removed) and balance was tested using only the vestibular system.

#### Other covariates

We assessed various potential covariates, including age, gender, race/ethnicity, education level, marital status, body mass index (BMI), poverty-income ratio (PIR), diabetes, and hypertension. Age was treated as a continuous variable. Race/ethnicity was categorized into five groups: Mexican American, other Hispanic, non-Hispanic white, non-Hispanic black, and other races. Education was categorized as less than high school, high school graduate, and more than high school. Marital status was categorized as married/cohabiting, separated/divorced, and single. PIR, which is the ratio of household income to the poverty line, was categorized as < 1 (below the poverty line) and ≥ 1 (at or above the poverty line). Participants were surveyed through questionnaires to inquire whether they had diabetes and hypertension. Having diabetes and hypertension was considered as having underlying diseases. BMI was categorized into three levels (normal: <25, overweight: 25–30, obese: >30).

#### Statistical analysis

Data were divided into continuous or categorical variables. Categorical variables were expressed as proportions (%), and continuous variables were expressed as means (standard deviations). T-tests or χ2 tests were used to compare the distribution of continuous or categorical variables. First, we compared the baseline characteristics of participants with and without vestibular dysfunction. Next, we conducted multiple regression analyses. In the multiple logistic regression models, we first established an unadjusted model (Model 1), then an adjusted model considering age, gender, and race/ethnicity (Model 2). Finally, we calculated a fully adjusted model (Model 3) using variables such as age, gender, race/ethnicity, education level, marital status, poverty-income ratio, and underlying diseases. Model 1 serves as the basic model, providing the unadjusted original associations; Model 2 adjusts for basic demographic variables to reduce the confounding effects of these fundamental characteristics; Model 3 makes further adjustments to comprehensively control for the potential confounding of lifestyle and socioeconomic factors, ensuring the robustness and reliability of the results. All statistical analyses were performed using R software (http://www.R-project.org) and EmpowerStats version 4.1 (http://www.empowerstats.net/analysis). Statistical significance was set at *p* < 0.05.

### Network pharmacology

#### Protein target identification

We used the PubChem (https://pubchem.ncbi.nlm.nih.gov/) and SwissTargetPrediction (http://www.swisstargetprediction.ch/) databases to screen for reported pharmacological targets of warfarin. Subsequently, we obtained the relevant targets of vestibular dysfunction from the GeneCards database (https://www.genecards.org/). In combination with the screening criteria related to vestibular dysfunction targets from other literature^19^ and the number of related targets, we set the inclusion criteria as a relevance score > 1. A Venn diagram was used to evaluate overlapping targets between warfarin and vestibular dysfunction.

#### Protein-protein interaction (PPI) network construction

Overlapping genes for warfarin and vestibular dysfunction were input into the STRING database (https://cn.string-db.org/) to establish a PPI network with a confidence score > 0.4. The interaction strength of the overlapping genes was analyzed.

#### KEGG pathway analysis

Using R software, we performed Kyoto Encyclopedia of Genes and Genomes (KEGG) pathway enrichment analysis on the overlapping targets of warfarin and vestibular dysfunction.

### Mendelian randomization analysis

#### Study design

First, we performed MR analysis on the exposure (warfarin use) and outcome (vestibular dysfunction). Next, we conducted MR analysis to validate the impact of the overlapping genes identified above on vestibular dysfunction.

#### Dataset description and Mendelian randomization analysis

All data for MR analysis were from publicly available genome-wide association studies (GWAS) and were derived. The GWAS dataset for warfarin use came from the UK Biobank, including 3,404 samples using warfarin and 333,755 UK descent control samples (GWAS ID: ukb-a-160). We used the FinnGen dataset for vestibular dysfunction GWAS (GWAS ID: finngen_R10_H8_VERTIGO)^20^, including data from 16,443 cases and 392,202 European descent controls, and this dataset is derived from the Finnish population.

First, we conducted a two-sample MR analysis of warfarin use and vestibular dysfunction (with a relaxed p-value threshold of 5*10 − 6 due to the small number of included SNPs). We used inverse variance weighted (IVW), MR-Egger, simple mode, weighted mode, and weighted median methods to evaluate the results, with IVW as the primary analysis method. The main analysis method was IVW, which is applicable in most situations. It assumes that all genetic instruments are valid, i.e., they only affect the outcome through the exposure and not through other pathways. MR-Egger was used to check for horizontal pleiotropy^21^. Simple Mode was employed to assess the consistency among genetic instruments, making it suitable for scenarios with a limited number of instrumental variables. Weighted Mode took into consideration the weight of each instrumental variable, rendering it appropriate for situations with a larger number of instrumental variables. Weighted Median demonstrated strong robustness against outliers and horizontal pleiotropy, also fitting for cases with a greater quantity of instrumental variables. Simple Mode, Weighted Mode, and Weighted Median were utilized to verify the reliability and stability of the results^22^. We assessed horizontal pleiotropy and heterogeneity to further increase the reliability of the study results. Finally, to further assess the robustness of the MR results, we conducted single-SNP analysis and leave-one-out analysis. Single-SNP analysis evaluated the impact of each individual genetic instrumental variable on the outcome, while leave-one-out analysis assessed the stability of the MR results by excluding each SNP one at a time.

Next, we performed an eQTL analysis on the overlapping genes identified above with vestibular dysfunction, using SNPs from European GWAS datasets^23^ as genetic instruments. Gene symbols were converted to ENSEMBL IDs to ensure consistency during the analysis. The p-value threshold for eQTL was set at 5*10 − 8. SNPs associated with the overlapping genes were extracted from the vestibular dysfunction GWAS dataset as instrumental variables for MR analysis. Finally, the TwoSampleMR package was used for MR analysis of these instrumental variables. If only one eQTL was available for a given overlapping gene, the Wald ratio represented the analysis result. When two or more genetic instruments were available, IVW was used as the primary analysis result. A p-value of less than 0.05 for IVW or Wald analysis indicated a significant association.

The version of R Studio used for analysis was 4.3.1, and the version of the TwoSampleMR package was 0.6.1.

### Molecular docking

Based on the above results, warfarin was used as the ligand, and genes with significant results from the MR analysis were used as receptors for molecular docking. The target protein structures were obtained from the Protein Data Bank (PDB) (https://www.rcsb.org/)^24^, and the 3D structures of the active components were downloaded from PubChem CID (https://pubchem.ncbi.nlm.nih.gov/) and UniProt databases. These were imported into AutoDock Vina, a plugin of PyMOL, to find the optimal conformation^25^.

## Results

### NHANES

**Fig. 2:**
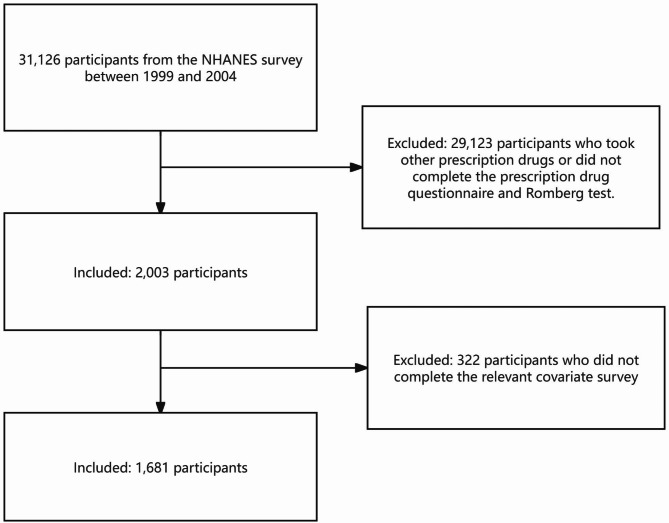
Flow chart of NHANES study population screening.

The study included a total of 1681 participants from NHANES conducted between 1999 and 2004. The flow chart illustrates the screening process for the study population (Fig. 2). The baseline characteristics of the participants were compared between those with vestibular dysfunction and those with normal vestibular function (Table 1). The overall prevalence of vestibular dysfunction in the study population was 27.32%. Notably, 13.00% of participants who used warfarin experienced vestibular dysfunction, compared to only 2.12% of those who did not use any prescription drugs. This preliminary comparison suggests a higher prevalence of vestibular dysfunction among warfarin users. In addition, we analyzed the impact of age, gender, race, educational level, marital status, poverty - income ratio (PIR), BMI, as well as underlying diseases such as diabetes and hypertension on vestibular dysfunction. We found that age, educational level, and PIR are potential risk factors for vestibular dysfunction (*p* < 0.001). The incidence of vestibular dysfunction was higher among individuals who were older, had lower levels of education, and had a lower PIR. Furthermore, we also discovered that different marital statuses and the presence of underlying diseases were associated with a higher prevalence of vestibular dysfunction (*p* < 0.05).

**Table 1:** Overall participant weighted characteristics based on vestibular dysfunction grouping from the 1999–2004 National health and nutrition examination survey.

	Total	Vestibular dysfunction (No)	Vestibular dysfunction (Yes)	*P*-value
Total (%)	1681(100.00)	1103(72.68)	578(27.32)	
Age(SD)	51.47(50.77,52.18)	49.37(48.83,49.90)	57.08(55.09,59.06)	**< 0.0001**
Gender				0.75
Male (%)	646(40.09)	426(39.77)	220(40.94)	
Female (%)	1035(59.91)	677(60.23)	358(59.06)	
Race/Ethnicity				0.19
Mexican American (%)	436( 6.80)	289(6.79)	147(6.81)	
Other Hispanic (%)	75( 5.82)	40(5.01)	35(7.99)	
Non-Hispanic White (%)	799(72.97)	530(74.57)	269(68.71)	
Non-Hispanic Black (%)	322(10.21)	212( 9.79)	110(11.32)	
Other race - including multi-racial (%)	49( 4.20)	32(3.84)	17(5.18)	
Poverty income ratio				**< 0.001**
≥1 (%)	1411(89.43)	949(91.44)	462(84.10)	
<1 (%)	270(10.57)	154(8.56)	116(15.90)	
Education level				**< 0.0001**
Below high schoo l(%)	588(19.49)	333(15.58)	255(29.88)	
High school (%)	366(24.29)	231(23.35)	135(26.79)	
Above high school (%)	727(56.22)	539(61.07)	188(43.33)	
Underlying diseases				**0.03**
No (%)	1334(82.05)	908(84.12)	426(76.55)	
Yes (%)	347(17.95)	195(15.88)	152(23.45)	
BMI				0.15
<25 (%)	528(33.46)	318(32.40)	210(36.29)	
25-30 (%)	690(40.19)	456(39.67)	234(41.55)	
>30 (%)	463(26.35)	329(27.92)	134(22.16)	
Marital status				**0.02**
Married or cohabiting (%)	1165(72.98)	795(74.95)	370(67.73)	
Divorce, Widower, Separated (%)	391(20.57)	234(19.21)	157(24.19)	
Never married (%)	125( 6.46)	74(5.84)	51(8.08)	
Warfarin use				**< 0.0001**
No (%)	1565 (94.91)	1068 (97.88)	497 (87.00)	
Yes (%)	116 (5.09)	35 (2.12)	81 (13.00)	

Significant values are in bold.

To assess the independent association between warfarin use and vestibular dysfunction, we conducted multiple logistic regression analyses. The results are presented in Table 2,In the unadjusted model(Model 1), warfarin use was significantly associated with vestibular dysfunction (OR = 6.89, 95% CI = 4.48–10.60, *p* < 0.000001). This indicates a strong raw association between warfarin use and the prevalence of vestibular dysfunction. After adjusting for age, gender, and race/ethnicity(Model 2), the association between warfarin use and vestibular dysfunction remained significant (OR = 2.28, 95% CI = 1.46–3.54, *p* < 0.001). This suggests that even when accounting for these basic demographic factors, warfarin use is still a significant predictor of vestibular dysfunction. In the fully adjusted model(Model 3), which included additional variables such as education level, marital status, PIR, underlying diseases, and BMI, the association between warfarin use and vestibular dysfunction continued to be significant (OR = 2.37, 95% CI = 1.41–4.00, *p* = 0.002). This indicates that the association is robust even after controlling for a wide range of potential confounding factors.

**Table 2:** Odds ratio (95% confidence Interval) between warfarin use and vestibular dysfunction among overall participants, National health and nutrition examination survey 1999–2004.

Characteristics	Model 1		Model 2		Model 3	
	OR (95% CI)	P-value	OR (95% CI)	P-value	OR (95% CI)	P-value
Warfarin use						
No	Reference		Reference		Reference	
Yes	6.89 (4.48,10.60)	<0.000001	2.28 (1.46,3.54)	<0.001	2.37 (1.41,4.00)	0.002

Model 1: Non-adjusted model. Model 2 was adjusted for sex, age in years, and race. Model 3 adjusted for the factors included in Model 2 and additionally adjusted for factors including education, marital status, poverty income ratio, underlying diseases, BMI.

### Network pharmacology

**Fig. 3:**
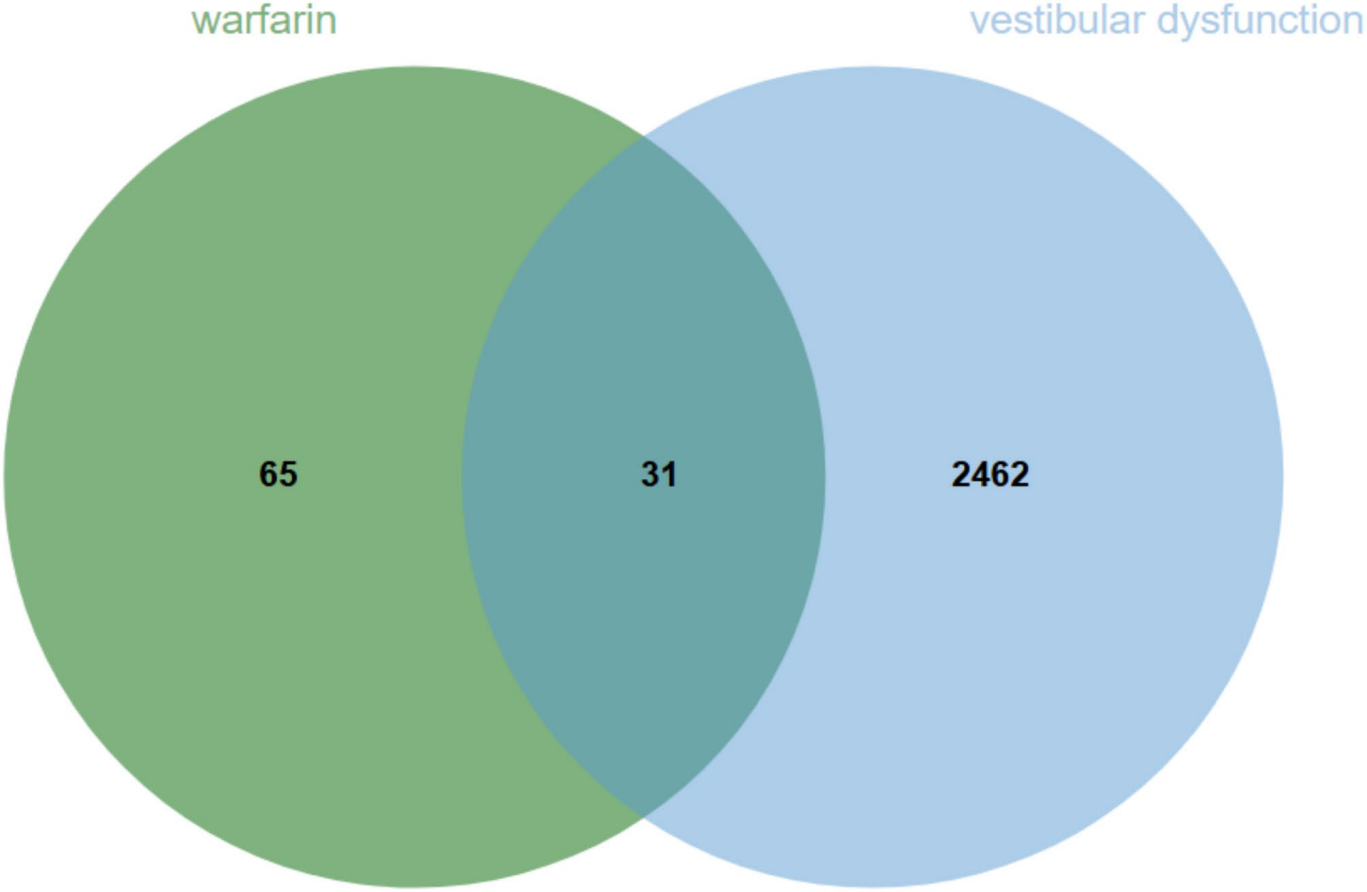
Warfarin and vestibular function key genes Venn diagram.

We screened 97 warfarin-related action genes from the PubChem and SwissTargetPrediction databases (Supplementary Materials 1). Subsequently, we collected 2,493 potential targets related to “vestibular dysfunction” from the GeneCards database with a relevance score > 1 (Supplementary Materials 2). By matching warfarin-related action genes with vestibular dysfunction-related genes, we identified 31 overlapping targets (Supplementary Materials 3), which are potential key genes for warfarin’s impact on vestibular function (Fig. 3).

**Fig. 4:**
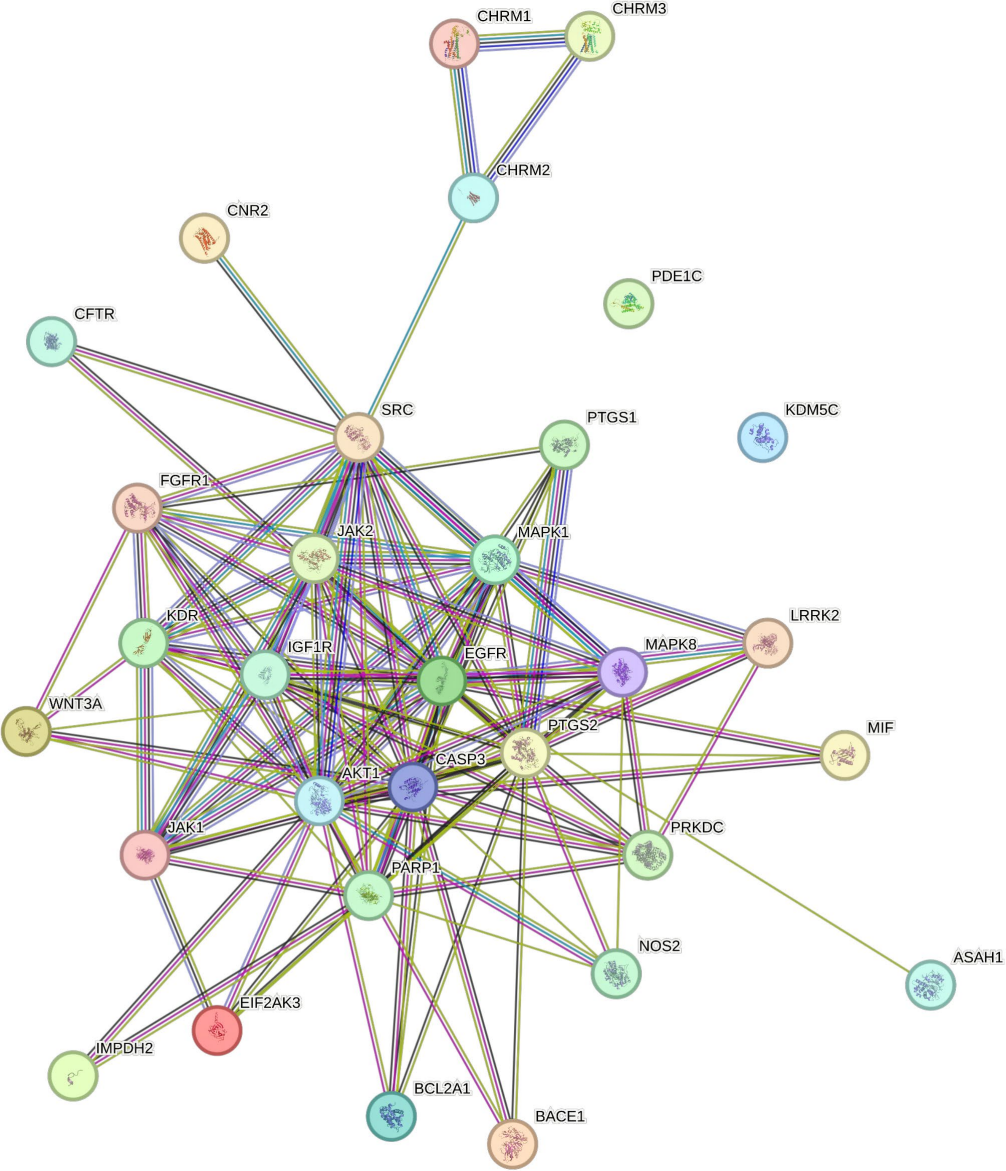
Warfarin and vestibular function overlap gene protein-protein interaction network diagram.

Using the STRING website, we created a PPI network diagram for the 31 overlapping genes and determined their interaction strengths (Fig. 4, Supplementary Materials 4).

**Fig. 5:**
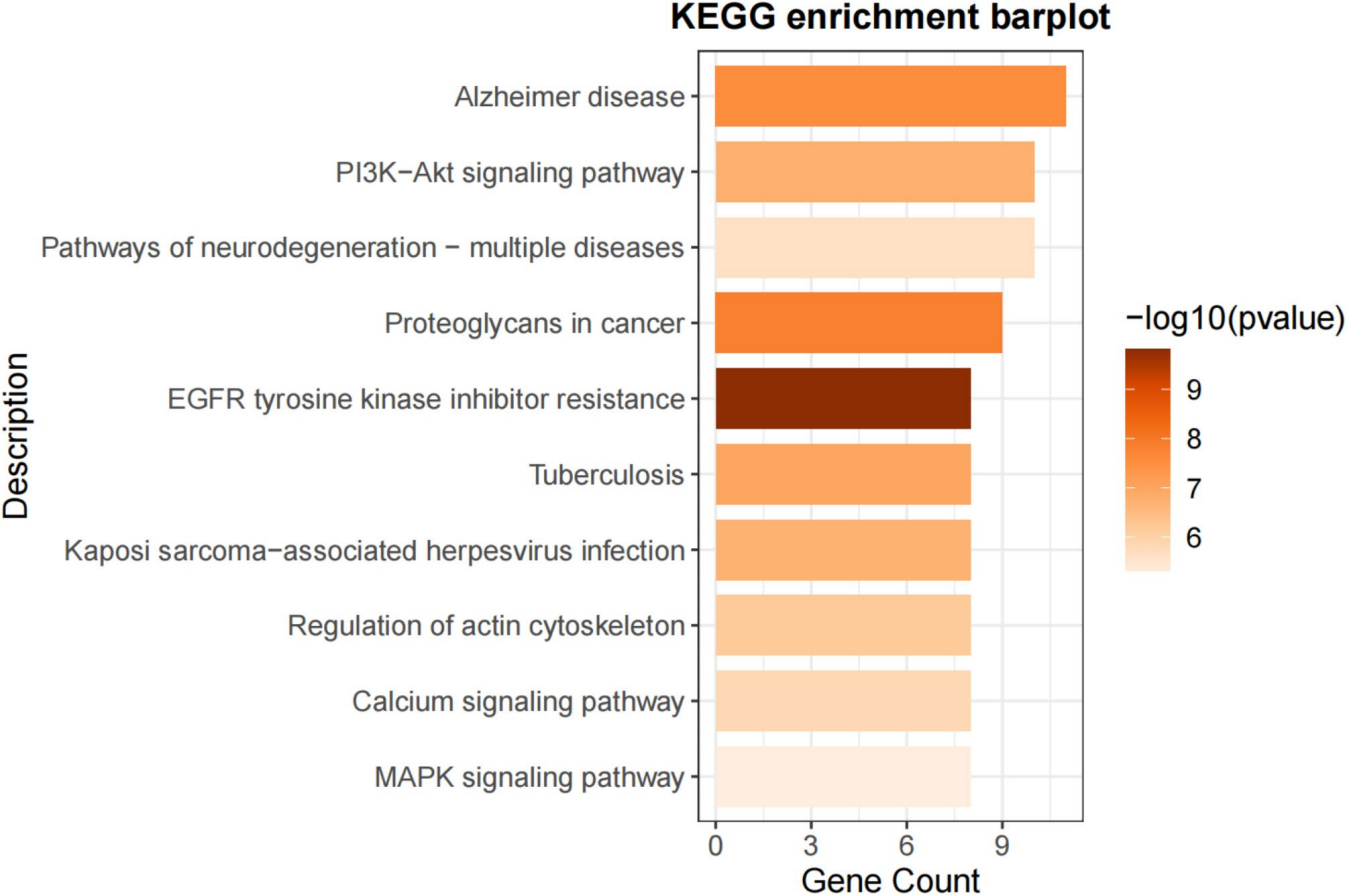
Results of KEGG analysis of overlapping genes.

KEGG pathway analysis of the 31 overlapping genes revealed the top 10 related pathways: Alzheimer’s disease, PI3K-Akt signaling pathway, pathways of neurodegeneration - multiple diseases, proteoglycans in cancer, EGFR tyrosine kinase inhibitor resistance, tuberculosis, Kaposi sarcoma-associated herpesvirus infection, regulation of actin cytoskeleton, calcium signaling pathway, and MAPK signaling pathway (Fig. 5).

### Mendelian randomization analysis

**Fig. 6:**
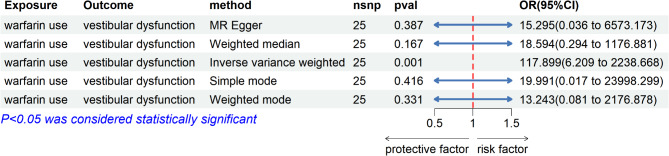
Forest plot of results from MR analysis of exposure (warfarin use) and outcome (vestibular dysfunction).

We performed MR analysis on the exposure (warfarin use) and outcome (vestibular dysfunction) using 25 eligible SNPs (Supplementary Materials 5). The results suggested a significant association, with IVW (OR = 117.898, 95% CI: 6.209-2.239E + 03, *P* = 0.001, Q_pval = 0.798) showing significance (Fig. 6). No horizontal pleiotropy (0.459) or heterogeneity (0.798) was detected (*P* > 0.05). The MR-Egger intercept was 0.005. The results of the leave-one-out analysis and single-SNP analysis are presented in the supplementary material (Supplementary Material Figs. 1, 2).

**Fig. 7:**
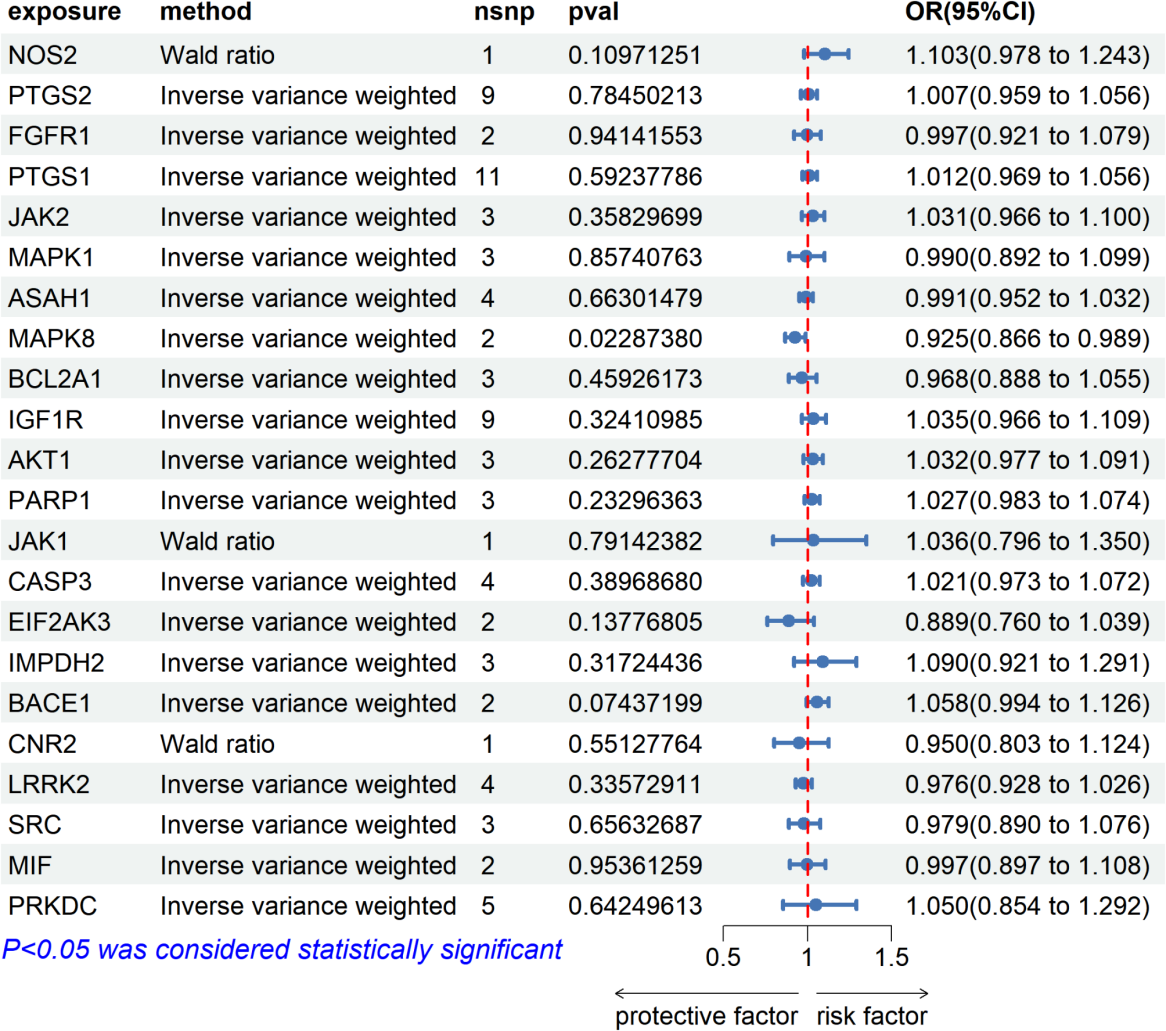
Forest plot of the results of Mendelian randomization analysis of overlapping genes and vestibular dysfunction.

MR analysis of overlapping genes and vestibular dysfunction indicated a significant association between MAPK8 and vestibular dysfunction (OR = 0.93, 95% CI: 0.87–0.99, *P* = 0.022), suggesting MAPK8 as a protective target for vestibular dysfunction (Fig. 7, Supplementary Materials 6). Due to the limited number of SNPs included, we did not perform analyses for horizontal pleiotropy and heterogeneity.

### Molecular docking

**Fig. 8:**
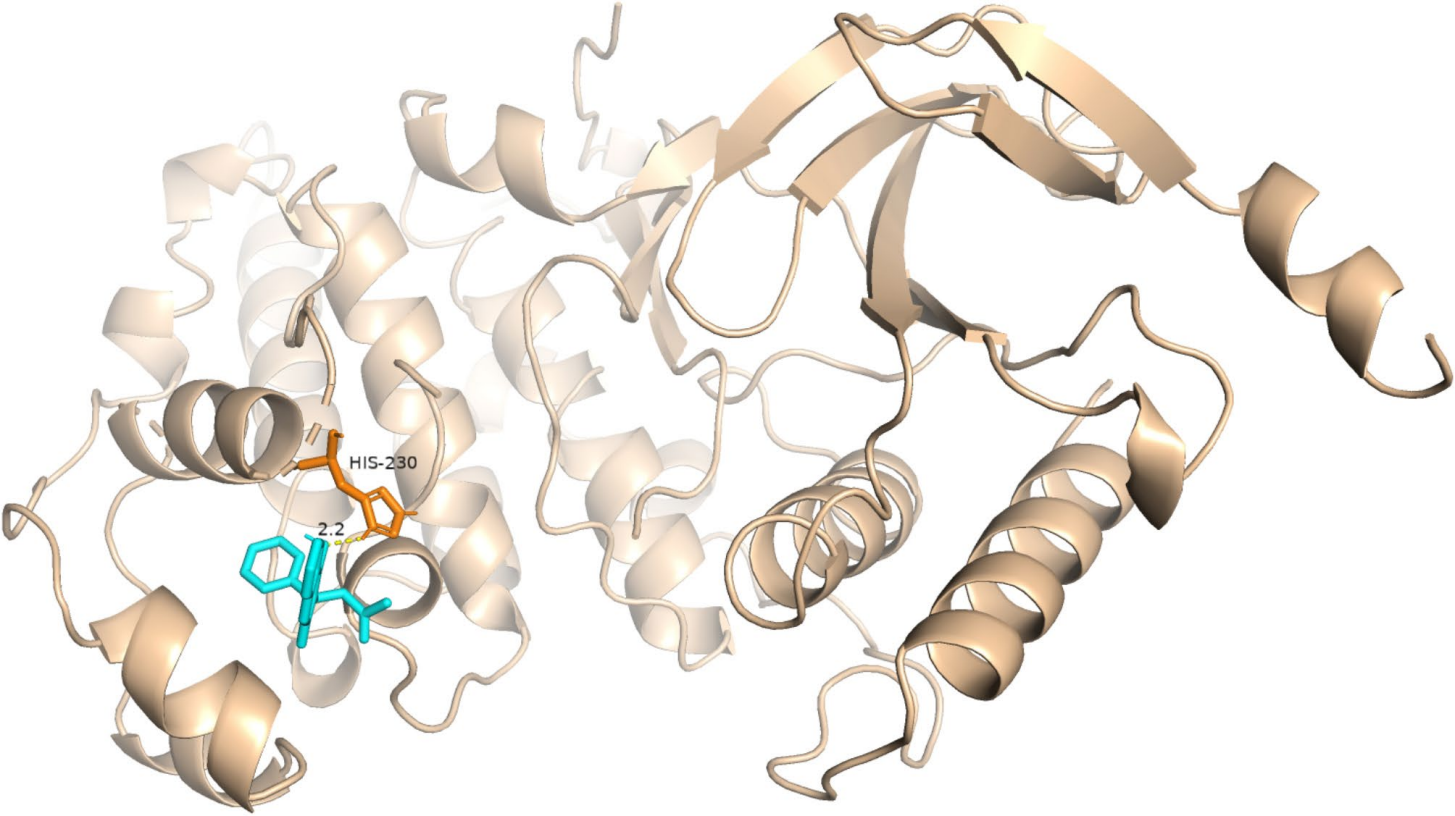
Warfarin and MAPK8 molecular docking results.

After reviewing the relevant literature, we selected the protein structure of MAPK8 (ID: 2xrw) from the PDB database for analysis^26,27^. Molecular docking between warfarin and MAPK8 was performed. The docking results showed a binding energy of -6.126 kcal/mol, indicating a good binding interaction between warfarin and MAPK8 (Fig. 8).

## Discussion

In this study, we conducted a cross-sectional analysis using the NHANES database to investigate the relationship between warfarin use and vestibular dysfunction. Our findings indicate that individuals taking warfarin are more likely to experience vestibular dysfunction. The MR analysis, which is a method that uses genetic variants as instrumental variables to infer potential causal relationships between an exposure and an outcome, suggested a significant positive relationship between warfarin use and vestibular dysfunction. This provides further support for the association observed in our cross-sectional analysis, although it is important to note that MR does not establish definitive causality.

Previous studies have not explored the impact of warfarin on vestibular function. Prior research primarily focused on warfarin’s potential effects on cognitive function^6,7^ or its association with other health risks^28,29^. Mechanistic studies have suggested that warfarin might impair the nervous system by affecting vitamin K activity^9^. Recent research also indicates that insufficient vitamin K intake may lead to dizziness or vertigo^30^. Since vestibular function is related to cognitive function and reflects the status of the nervous system to some extent^31–33^, the potential impact of warfarin on vestibular function warrants further investigation.

Our KEGG pathway analysis revealed that the overlapping genes affected by warfarin and associated with vestibular dysfunction are involved in pathways related to neurological diseases, the PI3K-Akt signaling pathway, and the MAPK signaling pathway. This suggests that warfarin may influence vestibular function through these pathways. MR analysis further indicated a significant association between MAPK8 and vestibular dysfunction, with MAPK8 potentially serving as a protective target. Molecular docking analysis demonstrated a good binding affinity between warfarin and MAPK8, suggesting that warfarin may directly affect MAPK8 activity, thereby influencing vestibular system function.

In the nervous system, MAPK8 activation is linked to various neurodegenerative diseases, playing a crucial role in intracellular signaling pathways^34–36^. Therefore, abnormal activation or inhibition of MAPK8 may also be related to the occurrence of vestibular dysfunction. The specific mechanisms by which MAPK8 is involved in warfarin-induced vestibular dysfunction are not entirely clear. Possible mechanisms include warfarin binding to MAPK8’s active site, altering its kinase activity, affecting downstream signaling, or influencing MAPK8 expression levels or stability, thereby disrupting its normal function in the vestibular system.

These findings need to be validated in broader populations. Future research should explore the association between MAPK8 gene polymorphisms and vestibular dysfunction and evaluate the impact of warfarin use on MAPK8 expression and activity. Additionally, our KEGG pathway analysis suggests that the MAPK pathway might play a key role in warfarin’s impact on vestibular function. Research has shown that the MAPK pathway and vitamin K may interact^37^, with MAPK8 being a critical gene in the MAPK pathway, potentially regulating vestibular function.

From a clinical perspective, the findings of this study suggest that clinicians should consider monitoring vestibular function in patients undergoing warfarin therapy, especially in those with known risk factors. Early detection of vestibular dysfunction can aid in timely intervention and management. Additionally, the potential role of genetic factors, such as MAPK8 gene variations, underscores the importance of personalized medicine. Genetic testing may help identify patients who are more susceptible to vestibular dysfunction during warfarin therapy, thereby enabling the development of more personalized treatment plans. For patients who experience vestibular dysfunction, clinicians may need to re-evaluate the risks and benefits of warfarin therapy and consider alternative anticoagulation strategies. Future research should validate these findings in more diverse populations to ensure the generalizability of the results. Prospective cohort studies will help establish a causal relationship between warfarin use and vestibular dysfunction and collect detailed information on dosage, duration, and adherence to better understand the dose-response relationship. Further mechanistic studies should explore the exact pathways through which warfarin affects vestibular function, particularly the role of MAPK8 and other identified genes. Future research should also focus on identifying diagnostic markers for early detection of vestibular dysfunction and potential therapeutic targets, such as modulators of the MAPK8 pathway, which could lead to new treatment strategies to mitigate the side effects of warfarin. Finally, interventional studies should test the effectiveness of potential treatments or interventions for preventing or managing vestibular dysfunction in patients on warfarin therapy, which may include pharmacological interventions, rehabilitation programs, or lifestyle changes.

The present study utilized MR to further investigate the cross-sectional NHANES study. However, the causal relationship between warfarin use and vestibular dysfunction remains to be fully elucidated. Detailed information regarding warfarin dosage and duration of use is still lacking, which limits our ability to assess dose-response relationships. Furthermore, warfarin use was determined by patients’ responses to the prescription drug use section of the NHANES survey, which is susceptible to reporting errors and may introduce bias. In addition, although the Romberg test is a traditional and effective method for detecting vestibular dysfunction, its efficiency is not 100%, and it may lead to false positives or false negatives, thereby affecting the accuracy of prevalence estimates. Therefore, future studies should include larger-scale prospective studies to clarify the impact of warfarin on vestibular function and explore its potential mechanisms. Secondly, the MR analysis used warfarin treatment as the exposure, which is inclined to be behavioral, and the genetic instrument may not fully capture the complex biological pathways involved. Lastly, although the data set of this study is from different countries, such as the United States, the United Kingdom, and Finland, which to some extent limits the wide generalizability of the research results, it is worth noting that we obtained similar analytical results in these different populations. This finding not only enhances the credibility of the research results but also indicates the universality and cross-population applicability of the study results.

In conclusion, our study suggests that warfarin may affect vestibular function through the MAPK8 target. This finding is clinically significant and opens new avenues for research into the mechanisms of vestibular dysfunction. Monitoring and preventing vestibular dysfunction during warfarin therapy, considering genetic backgrounds and MAPK8 variations, may be crucial for individualizing treatment and adjusting medication dosages.

## Electronic supplementary material

Below is the link to the electronic supplementary material.

Supplementary Material 1

Supplementary Material 2

## Data Availability

All the data can be obtained from the open source platform provided in the article. This study analyzed data from the database, NHANES database (https://www.cdc.gov/nchs/nhanes/index.htm), IEU database (https://gwas.mrcieu.ac.uk/), FINNGEN database (https://www.finngen.fi/en/access_results), PubChem (https://pubchem.ncbi.nlm.nih.gov/), SwissTargetPrediction (http://www.swisstargetprediction.ch/), GeneCards (https://www.genecards.org/), Protein data bank (https://www.rcsb.org/), PubChem CID (https://pubchem.ncbi.nlm.nih.gov/).
