# Supplementary material for: Warfarin use and vestibular dysfunction insights from NHANES data, network pharmacology, Mendelian randomization, and molecular docking
Source: Sci Rep. 2025 Apr 6;15:11748. doi: 10.1038/s41598-025-96681-5 (PMC11973179; doi:10.1038/s41598-025-96681-5)
Supplement: Supplementary file 1 — Supplementary Material 1 [file 41598_2025_96681_MOESM1_ESM.docx]

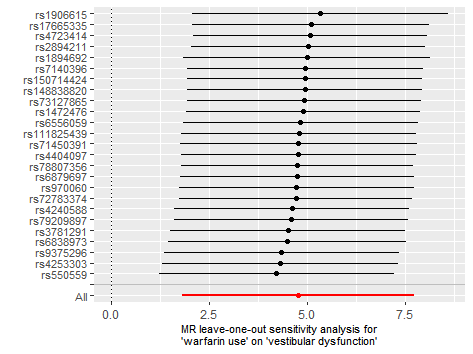


（Supplementary Material Figure 1: A leave-one-out analysis of the estimations for warfarin use and vestibular dysfunction.）


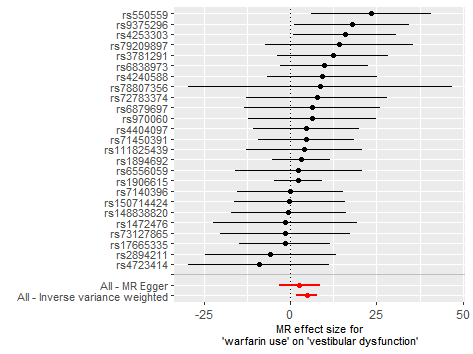
（Supplementary Material Figure 2: Forest plot of MR sensitivity analysis. The IVM method showed a MR Effect size greater than 0, indicating a causal effect of warfarin use on vestibular dysfunction.）
